# Warfarin-induced gastrointestinal bleeding with acute liver failure: A case report

**DOI:** 10.1097/MD.0000000000040658

**Published:** 2024-11-22

**Authors:** Jiahao Chen, Le Li, Shiyu Wang

**Affiliations:** a Department of Pharmacy, The People’s Hospital of Hezhou, Hezhou, China.

**Keywords:** gastrointestinal bleeding, liver failure, pharmacy monitoring, warfarin

## Abstract

**Rationale:**

Warfarin is the most commonly used drug in patients with mechanical valve replacement. Acute liver damage after warfarin is rare but potentially harmful. We present a case of warfarin-induced gastrointestinal bleeding with liver injury, pharmacy monitoring, and its therapy.

**Patient concerns:**

A 64-year-old woman with warfarin 4.5 mg medical history 10 years after mechanical mitral valve replacement. Who presented with gastrointestinal bleeding and extensive ecchymosis, due to rising international normalized ratio (INR), and then progressed to acute liver injury.

**Diagnoses:**

Warfarin poisoning.

**Interventions:**

Discontinuing warfarin, and artificial liver support system with anti-inflammatory liver therapy, which used reduced glutathione, polyene phosphatidylcholine, and ademetionine 1,4-butanedisulfonate for injection, ursodeoxycholic acid for orally.

**Outcomes:**

The liver enzymes and hyperbilirubinemia were improved, she was placed on warfarin again, and the INR increased to 2.03. There was no significant increase in liver enzymes and hyperbilirubinemia, she was discharged on day 24.

**Lessons:**

Close monitoring and immediate dose adjustment of warfarin and to avoid drug–drug interaction. Timely stopped warfarin, adjusted INR and anti-inflammatory liver therapy may reduce the occurrence of warfarin-induced liver failure.

## 1. Introduction

Warfarin is a coumarin anticoagulant, the only used drug for long-term anticoagulant therapy after mechanical mitral valve replacement. The most common adverse reaction of warfarin is hemorrhage, and thus, the international normalized ratio (INR) should be monitored and the dose should be adjusted timely in patients taking warfarin. Other minor adverse reactions of warfarin include nausea, diarrheal, and skin rash. Nevertheless, warfarin-induced acute liver injury has rarely been reported.

We reported a case of liver injury induced by warfarin, the liver enzymes and hyperbilirubinemia were improved after discontinuing warfarin, artificial liver support system with anti-inflammatory liver therapy.

## 2. Case report

**Figure 1.**
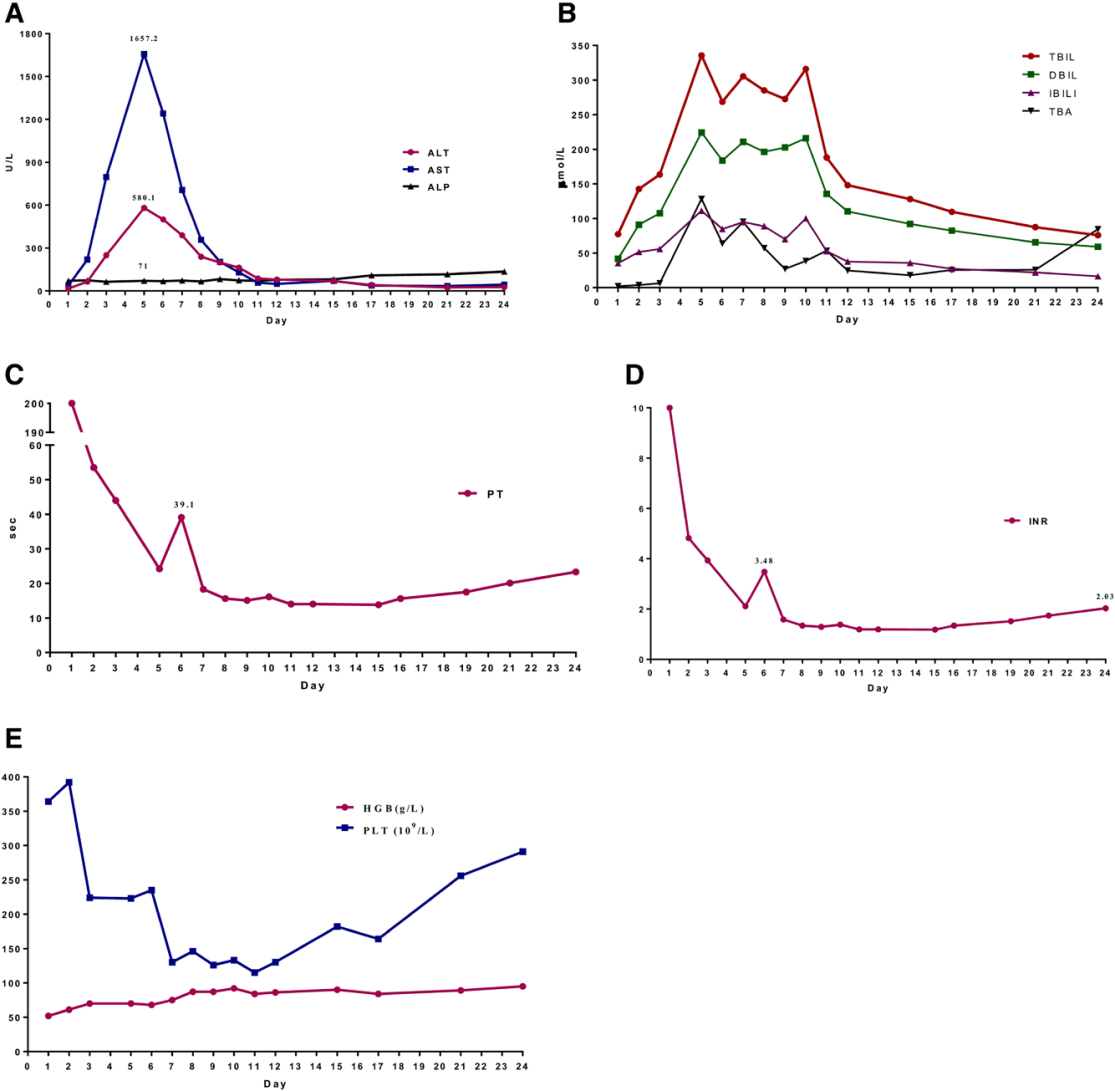
Laboratory tests including liver function, coagulation function, and routine blood tests.

A 64-year-old woman presented to our hospital with extensive ecchymosis and black stool, who was treated with warfarin 4.5 mg for 10 years after mechanical mitral valve replacement. Upon clinical suspicion of coagulation disorders, a comprehensive laboratory panel was performed, the INR of the patient exceeded 10.0, warfarin was discontinued and vitamin K1 10 mg by intravenous injection, then review the INR reduced to 5.53. Five days later the INR reduced to 1.83, discontinued vitamin K1, the bleeding was effectively controlled. But the liver enzymes and hyperbilirubinemia rose abnormally, the alanine transaminase (ALT), aspartate aminotransferase, total bilirubin, direct bilirubin, indirect bilirubin, and total bile acid levels were significantly increased (Fig. 1). The patient was prescribed glutathione, polyene phosphatidylcholine, and ademetionine 1,4-butanedisulfonate for injection, ursodeoxycholic acid for orally to protect the liver (the details of drug usage are listed in Table 1). Two days later the liver injury aggravated, and the patient was then transferred to the intensive care unit.

**Table 1:** Drug usage in hospital.

Drugs	Usage	Start time	Withdrawal times
Vitamin K1	10 mg	Day1	Day 4
		Day 6	Day 15
Glutathione	2.4 g qd	Day 3	Day 24
Polyene phosphatidylcholine	930 mg qd	Day 3	Day 24
Ademetionine 1,4-butanedisulfonate	1 g qd	Day 3	Day 24
Ursodeoxycholic acid	250 mg bid	Day 3	Day 24
Esomeprazole	40 mg bid	Day 1	Day 1
	80 mg bid	Day 2	Day 5
	40 mg bid	Day 6	Day 24
Ceftriaxone	2 g qd	Day 2	Day 21
Warfarin	2.5 mg qd	Day 16	Day 19
	3.75 mg qd	Day 20	Day 24
Furosemide	10 mg qd	Day 2	Day 3
	20 mg qd	Day 4	Day 4
	20 mg bid	Day 5	Day 15
Torasemide	10 mg qd	Day 16	Day 24
Antisterone	20 mg qd	Day 15	Day 24
Digoxin	0.125 mg qd	Day 19	Day 24

At the intensive care unit, the INR raised to 3.48, the liver enzymes and hyperbilirubinemia were further, and the patient received vitamin K1 10 mg by intravenous injection and artificial liver support system (qod, 5d). On hospital day 15, the patient's INR decreased to <2.0, the liver enzymes and hyperbilirubinemia were improved, she was placed on warfarin again, and the INR increased to 2.03. There was no significant increase in liver enzymes and hyperbilirubinemia, she was discharged on day 24.

## 3. Discussion

The liver injury that occurred in this case was thought to be related to warfarin. To our knowledge, our study reported the first case of warfarin-induced hepatocellular pattern of liver injury (extreme elevations in ALT and an ALT/alkaline phosphatase ≥5) in an adult. However, assigning drug causality is often difficult, especially when multiple agents are implicated and combine heart disease.

Acute heart failure may lead to liver congestion, the liver enzymes and hyperbilirubinemia may be rise. But this patient was no edema in both lower limbs and upright breathing, and the left ventricular was normal sized (left ventricular ejection fractions = 62%), when liver function damage progressed the NT-proBNP significantly declined (from 7237 to 3000 μg/L), it could eliminate the acute heart failure caused the liver injury.

Potential factors that might have contributed to the significant change in the patient’s liver function with warfarin included chronic liver, food, sex, age, and drug interaction. The results of hepatitis A, B, C, D, and E screening showed normally, excluded the chronic liver cause liver injury in this patient. Food was reported to interact with warfarin, while most food was safely taken in moderation, no new food types were found by asking the patient about her diet structure.

The differences in sex hormone levels and gene expression were the main variations in causes of the incidence of hepatic diseases,^[1]^ women more commonly present with acute liver failure, and drug-induced hepatotoxicity.^[2]^ The elevated blood concentrations and longer elimination times were manifested by women, and strongly linked to sex differences in potential hepatotoxicity.^[3]^ Aging has been shown to enhance vulnerability to acute liver injury,^[4]^ due to structural alteration or dysfunction. The decreased basal metabolic rate and renal blood flow could change the distribution and clearance of warfarin in older individuals. Then the other possible factor to the liver injury was the drug.

The other possible contributing factor to the liver injury was drug interaction. Warfarin is metabolized by the cytochrome P450 (CYP) isozymes in the liver, primarily by the CYP2C9, CYP2C19, CYP2C8, CYP1A2, and CYP3A4.^[5]^ Among the medications that the patient was receiving in the hospital, the suspected drugs are esomeprazole and ceftriaxone, both of them can elevate liver enzymes, and the esomeprazole interacts with warfarin are metabolized by CYP3A4 and CYP2C19.^[6]^ But as the liver injury improved, the warfarin retakes, the liver function was not affected, and this combination was not considered to be the cause of the liver failure. Therefore, we suspected warfarin overdose as the culprit drug.

What causes warfarin accumulation in the body? We asked the patient and her family about the detailed medication history, known she had taking herb for weakness and lower limb pain, though the specific drug was unclear. Some herbs that have been shown to interact with the CYP isozymes,^[7]^ which may cause warfarin accumulation and INR were abnormally elevated. Even though this drug interaction was categorized as possible or probable, we recommended that physicians and patients closely monitor the INR while taking warfarin and herbs, although there is insufficient evidence to link these interactions.

## 4. Conclusion

We present a case of liver injury, possibly due to warfarin. Close monitoring INR and timely stopped warfarin and to avoid drug–drug interaction, artificial liver support system with anti-inflammatory liver therapy may reduce the occurrence of warfarin-induced liver failure.

## Author contributions

**Data curation:** Jiahao Chen, Le Li.

**Writing—original draft:** Jiahao Chen.

**Investigation:** Le Li.

**Writing—review & editing:** Shiyu Wang.
